# A silent two-photon imaging system for studying in vivo auditory neuronal functions

**DOI:** 10.1038/s41377-022-00783-y

**Published:** 2022-04-14

**Authors:** Xindong Song, Yueqi Guo, Chenggang Chen, Xiaoqin Wang

**Affiliations:** Laboratory of Auditory Neurophysiology, Department of Biomedical Engineering, Johns Hopkins University School of Medicine, Baltimore, MD 21205 USA

**Keywords:** Multiphoton microscopy, Ca2+ imaging, Biophotonics

## Abstract

Two-photon laser-scanning microscopy has become an essential tool for imaging neuronal functions in vivo and has been applied to different parts of the neural system, including the auditory system. However, many components of a two-photon microscope, such as galvanometer-based laser scanners, generate mechanical vibrations and thus acoustic artifacts, making it difficult to interpret auditory responses from recorded neurons. Here, we report the development of a silent two-photon imaging system and its applications in the common marmoset (*Callithrix Jacchus*), a non-human primate species sharing a similar hearing range with humans. By utilizing an orthogonal pair of acousto-optical deflectors (AODs), full-frame raster scanning at video rate was achieved without introducing mechanical vibrations. Imaging depth can be optically controlled by adjusting the chirping speed on the AODs without any mechanical motion along the Z-axis. Furthermore, all other sound-generating components of the system were acoustically isolated, leaving the noise floor of the working system below the marmoset’s hearing threshold. Imaging with the system in awake marmosets revealed many auditory cortex neurons that exhibited maximal responses at low sound levels, which were not possible to study using traditional two-photon imaging systems. This is the first demonstration of a silent two-photon imaging system that is capable of imaging auditory neuronal functions in vivo without acoustic artifacts. This capacity opens new opportunities for a better understanding of auditory functions in the brain and helps isolate animal behavior from microscope-generated acoustic interference.

## Abstract (web summary)

Silent two-photon imaging is demonstrated with non-mechanical AOD scanners. Video-rate recordings in auditory cortex of awake marmoset monkeys reveal neuronal functions at unprecedented low sound levels.

## Graphical abstract

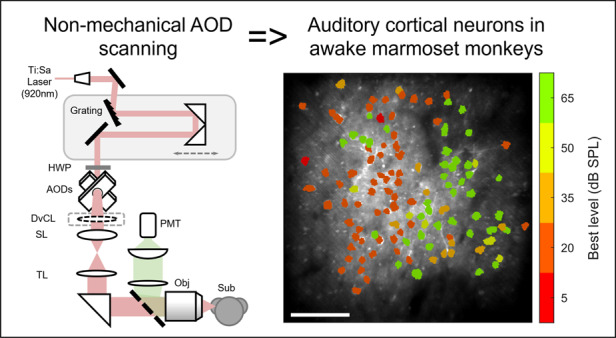

## Introduction

Two-photon laser-scanning microscopy (TPLSM) has revolutionized the way that neuroscientists study functions in neuronal populations. Its applications have been widely adopted for a variety of brain regions and functional modalities e.g., visual system^1^, somatosensory system^2^, and motor system^3^. However, applying the TPLSM method to the auditory system is technically challenging. Because many components of a TPLSM system undergo mechanical movements, acoustic noises are generated. For example, galvanometer-based mirror scanners produce acoustic power with spectral peaks at distinct frequencies depending on the scanning pattern (e.g., a resonant galvanometer scanner of 8 kHz was reported generating ~45 dB SPL sound at the location of the experimental animal’s head^4,5^); a Ti:Sapphire laser system generates substantial broadband noise with a bias towards low frequencies at its power supply and cooling units. Although efforts have been made to isolate an experimental subject from some of the noise sources^6–12^, to date, there is neither a TPLSM system specifically designed with a non-mechanical scanning mechanism to keep silent in auditory experiments nor an established way to assess imaging noise with respect to the experimental species’ hearing sensitivity.

**Fig. 1:**
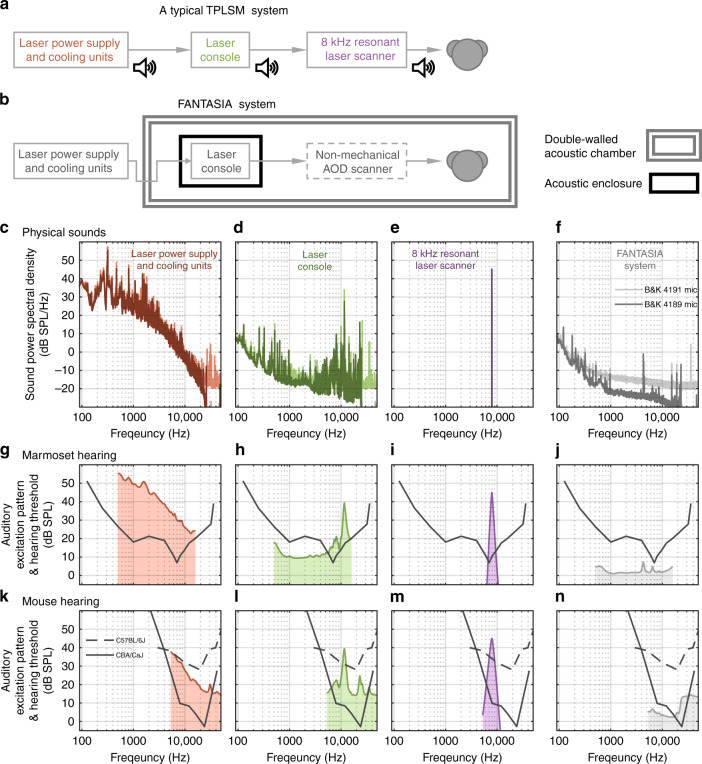
Acoustic noises of TPLSM systems. **a** An illustration of major sound-generating components in a typical TPLSM system. **b** The strategies for eliminating two-photon imaging sounds in our FANTASIA system. **c**–**f** The acoustic power spectral densities of imaging noises. These noises include the sounds from several components of a typical TPLSM system (**c**–**e**) and the operating noise of our FANTASIA system (**f**). Each sound was measured twice by two different calibration microphones (B&K 4189 with a standard spectral range and a low noise floor; B&K 4191 with an extended spectral range and an elevated noise floor). **g**–**j** The auditory excitation patterns are generated from the noises in (**c**–**f**). These excitation patterns are estimated in marmoset monkeys, with the marmoset hearing threshold^17^ also plotted as a reference (black solid curve). **k**–**n** The mouse auditory excitation patterns are generated from the noises in (**c**–**f**), with the hearing thresholds of both CBA/CaJ strain^25^ and C57BL/6J strain^33^ also plotted as references (solid and dashed black curves)

**Fig. 2:**
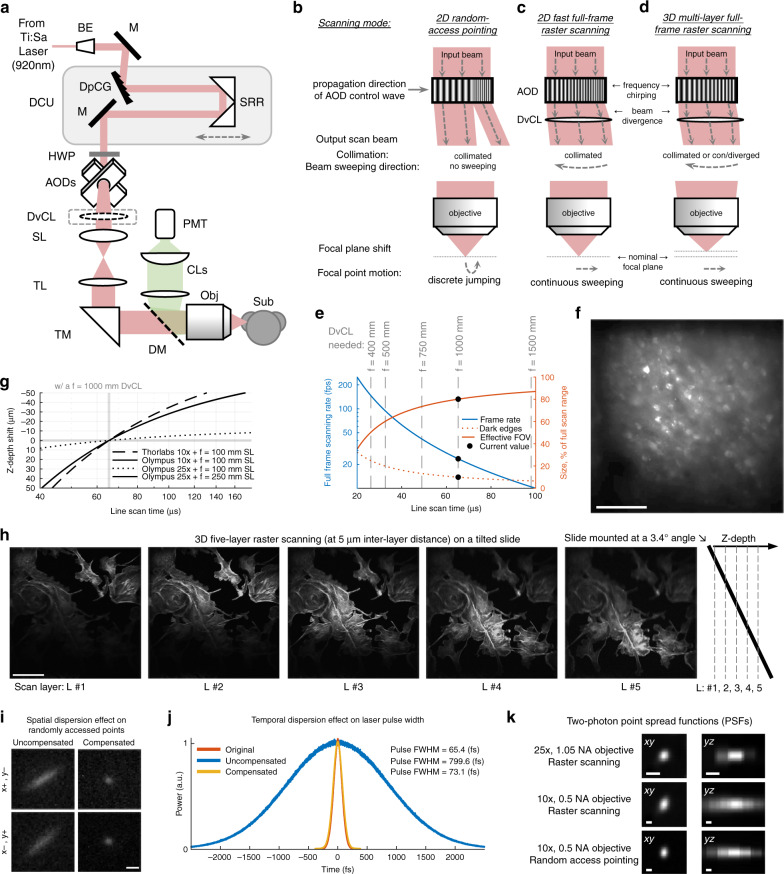
The optical design of the FANTASIA system. **a** A single-pair AOD based two-photon imaging system with a simple architecture. BE (beam expander); M (mirror); DpCG (dispersion compensation grating); SRR (sliding retroreflector); DCU (dispersion compensation unit); HWP (halfwave plate); AODs (acousto-optical deflectors); DvCL (divergence compensation lens); SL (scan lens); TL (tube lens); TM (turning mirror); DF (dichroic filter); Obj (objective); Sub (subject); CLs (collection lenses); PMT (photomultiplier tube). **b**–**d** Schematics of scanning modes supported by the system. A basic scanning mode supported by an orthogonal pair of AODs is 2D random-access pointing (**b**). The system further supports 2D fast raster scanning (**c**) by chirping the AOD control signals on both axes at the same speed, while the beam divergence generated by the AODs is compensated with an additional convex lens (DvCL). The focal plane depth can be additionally shifted by adjusting the chirping speed of the AOD control signals, resulting in a 3D multi-layer raster scanning mode (**d**). **e** The effect of AOD chirping speed on the frame rate, the DvCL needed, and the FOV size in the 2D fast full-frame raster scanning mode in (**c**). The AOD chirping speed is expressed in line scan time, the time to linearly chirp across the AOD bandwidth (30 MHz). A shorter line scan time would result in a higher full-frame raster scanning rate, a need for a DvCL with a shorter focal length to fully compensate for beam divergence, and a wider dark periphery in the FOV due to the necessary transition time between the scanning lines. **f** Two-photon image acquired with the 2D fast full-frame raster scanning mode demonstrated in (**c**). The image is of a neuronal population labeled with GCaMP6s in marmoset auditory cortex at a ~200 μm depth. Scale bar: 100 μm. **g** The effect of AOD chirping speed on focal depth shift in the 3D multi-layer full-frame raster scanning mode (**d**) with an f = 1000 mm DvCL. The AOD chirping speed is expressed in line scan time as in (**e**). This effect is also DvCL- and objective- dependent. **h** Two-photon images acquired with the 3D multi-layer raster scanning mode demonstrated in (**d**). The five scanned layers are spaced by a 5 μm inter-layer distance. The images are of a testing slide (Invitrogen F36924) tilted at a 3.4° angle (~25 μm depth change over the FOV). Scale bar: 100 μm. **i** The spatial dispersion effect demonstrated by wide-field images of two randomly accessed spots pointed on a fluorescence reference slide (Ted Pella 2273) without (uncompensated) and with (compensated) the DCU. These two points are at the opposite corners of the FOV (x-, y+ and x+ , y-). Scale bar: eq. 1 mrad of AOD scanning angle. **j** The temporal dispersion effect on laser pulse width. The autocorrelation functions were measured at the laser output (original) and after the AODs without (uncompensated) and with (compensated) the DCU. Pulse width estimation is based on sech^2^ deconvolution of 0.65 times autocorrelation. **k** The two-photon point spread functions (PSFs) of the system. The PSFs were measured with the Thorlabs 10x, 0.5 NA objective on 0.5-µm microbeads (1^st^ and 2^nd^ row) and with the Olympus 25x, 1.05 NA objective on 0.2-µm microbeads (3^rd^ row). Measurements were taken under random access pointing mode (1^st^ row) and raster scanning mode (2^nd^ and 3^rd^ row). Scale bar: 1 μm. FWHM (lateral, axial): 1.17 ± 0.22 µm, 6.38 ± 0.19 µm (1^st^ row); 1.45 ± 0.23 µm, 7.78 ± 0.65 µm (2^nd^ row); 0.56 ± 0.06 µm, 1.42 ± 0.04 µm (3^rd^ row)

Among non-mechanical laser-scanning mechanisms, acousto-optical deflectors (AODs) are a good candidate for designing a silent high-performance TPLSM. They employ solid-state ultrasonic waves across the AOD crystals at frequencies (~100 MHz) that are ~1000 times higher than what mammals can hear to deflect laser beams in a fast fashion^13,14^. Recent studies have utilized AODs in multiple pairs to achieve high flexibility in scanning modes^15,16^ (e.g., 3D random-access pointing, continuous lines, surfaces, etc.). However, the architectural complexity in these multi-pair AOD systems raises the technical difficulty for others to replicate and access these systems. Here we developed a simplified single-pair AOD based TPLSM system that can achieve a high degree of scanning flexibility while remaining agile in scanning speed. Our system can quickly switch among scanning modes of 2D random-access pointing (up to 50k points/second); 2D full-frame raster scanning (up to 23.5 frames/second); and 3D multi-layer raster scanning across different depths (Fig. 2). Additionally, acoustic treatment was performed throughout the system. The final system features a low acoustic noise floor during imaging (<25.9 dB SPL within the 100-40,000 Hz band, Fig. 1).

We further applied our system to image neuronal functions in the auditory cortex of common marmosets (*Callithrix Jacchus*), a non-human primate (NHP) species that shares many basic and higher-order auditory attributes with humans (e.g., hearing range and sensitivity;^17^ human-like complex pitch perception mechanisms^18^). Unlike most other NHP models, the marmoset auditory cortex is located primarily on the brain surface outside the lateral sulcus, leading to a unique opportunity to study auditory cortical functions using optical imaging techniques^19–22^. In awake marmosets, our TPLSM system resolved clear neuronal structures such as somas and processes, while robust functional tunings were mapped out at sound levels that were previously not feasible in two-photon imaging studies due to acoustic noises. Many recorded neurons showed level-dependent frequency sensitivity with their maximal responses at low sound levels (e.g., 20 dB SPL), suggesting the utility of our system for delicate auditory studies.

Moreover, to evaluate the acoustic noise of an imaging system, we modified a loudness model^23^ to account for species-dependent frequency selectivity and hearing sensitivity. With the model, the power spectral density of an acoustic noise can be converted into a species-dependent auditory excitation pattern and then compared to the species’ hearing threshold. We demonstrated that the operating noise of our TPLSM system generates an excitation pattern that is near or below the hearing threshold in many species (e.g., marmosets, mice, ferrets, rats), suggesting a clean acoustic background for auditory studies in these species.

Together, we demonstrated a “Flexible, Agile, and auditorily Noise-free Two-photon AOD Scanning Imaging system for use in Awake animals” (termed FANTASIA) and made the design and related code publicly available. The FANTASIA system opens new opportunities for sensitive experiments that require minimal acoustic interference from the two-photon imaging apparatus, both perceptually and behaviorally. The procedures that we established to assess the acoustic noise of an imaging system can also be generalized to other setups and species. This easily adaptable approach provides auditory two-photon imaging studies with improved clarity and data interpretability and will likely lead to a better understanding of brain functions.

## Results

### Quantifying acoustic noises of TPLSM components

To evaluate acoustic noises, sounds from several TPLSM components (Fig. 1a) were quantified by either direct measurements or existing data from the literature. These sound power spectra are shown in Fig. 1c–f (in dB SPL/Hz). The noise of the Ti:Sapphire laser’s power supply and cooling units was measured and plotted in Fig. 1c. The power spectrum is broadband and features a plateau (20-50 dB SPL/Hz) mainly at frequencies below 1 kHz. The power spectrum gradually decreases with increasing frequency and drops below 0 dB SPL/Hz when the frequency goes above 10 kHz. This noise also appeared loud to human operators nearby. We thus separated the laser power supply and cooling units outside a double-wall acoustic chamber (Fig. 1b). Cooling lines and connections to the laser console entered the chamber via U-shaped PVC tubes whose remaining gaps were filled with acoustic foam. Interestingly, after the separation, a sound from the laser console became audible to human operators. This sound produced a bump and a few sharp peaks at a frequency range of 6-23 kHz in the spectrum (Fig. 1d). It is likely that some mechanical components (e.g., piezo actuators) are active within the Ti:Sapphire laser console during operation. This frequency range is within the hearing range of most mammalian species (Fig. S1). Particularly, marmoset monkeys are very sensitive to this spectral range^17,24^ and their vocalizations have acoustic power largely within this range^24^. Other species, such as CBA/CaJ mice, are also very sensitive to this range^25^. We customized a sound enclosure for the laser console to attenuate this high-frequency sound (Fig. 1b). Furthermore, the sound from laser scanners was reported to generate distinct spectral peaks in the power spectrum^4–10,12^. Modern TPLSMs demand fast scanning and frequently use a resonant scanner for raster scanning on the fast axis. It was reported that a resonant scanner with a resonant frequency of 8 kHz produced a sound of 45 dB SPL^4,5^. Again, many mammalian species have hearing thresholds near or below 10 dB SPL at this frequency^17,25–27^ (Fig. S1). Marmoset hearing threshold, for instance, was reported as 10.6 dB SPL^17^, suggesting using an 8-kHz resonant scanner, may introduce major acoustic artifacts to auditory experiments in species such as marmosets. It is thus desirable to design a non-mechanical scanning TPLSM (Fig. 1b) that does not actively generate acoustic power within the hearing range of common experimental species (Fig. S1). After these acoustic treatments, our imaging system “FANTASIA” was so quiet that the measurements of its operating sounds had reached, and were thus limited by, the electrical noise floors of calibration microphones (Fig. 1f, see also Materials and methods).

### Assessment of the TPLSM system’s noise floor with respect to the experimental species’ hearing

How loud is the imaging noise to an experimental species? Noises are typically broadband and quantified as power spectral densities in dB SPL/Hz (Fig. 1c–f), while the hearing thresholds are measured using pure tones varying in level (in dB SPL). Therefore, at a given frequency, the noise power spectral density needs to be integrated across a certain bandwidth to be compared to the hearing threshold. Indeed, psychoacoustic studies^28^ have revealed a “critical band for loudness” (CB_L_) that accounts for this loudness integration bandwidth or frequency selectivity in human hearing. We took this frequency selectivity part of a loudness model built upon human psychoacoustics^23^ and substitute human parameters with marmoset parameters^29^. The modified model would thus convert a physical power spectrum (in dB SPL/Hz) into a marmoset-specific “excitation pattern” (in dB SPL) that accounts for the frequency selectivity of the species. Any part of the excitation pattern well exceeding the species’ hearing threshold would suggest the sound may excite the species’ auditory system efficiently at the corresponding frequency. Figure 1g–j show how noises from TPLSM systems generate auditory excitation patterns in marmosets. Marmoset hearing threshold^17^ was plotted in a black solid line as a reference. The untreated laser power supply and cooling units generate an excitation pattern constantly above the hearing threshold by roughly 20 dB from the low-frequency end to around 7 kHz (Fig. 1g). Moreover, the untreated laser console generates an excitation pattern that exceeds the hearing threshold between 5 kHz and 16 kHz by up to more than 20 dB (Fig. 1h). Furthermore, an 8-kHz resonant scanner may generate an excitation pattern with a peak at the most sensitive frequency region in marmosets (Fig. 1i). In contrast, our FANTASIA system features a noise floor with its excitation pattern below the marmoset hearing threshold across the entire frequency range (Fig. 1j). In addition to marmosets, the same analysis was repeated for mice (Fig. 1k–n) and other species (Fig. S1). In mice, two-photon studies were frequently performed in animals with C57BL/6 genetic background^4–10,30^, which naturally develop progressive hearing loss as early as 7 weeks postnatal^11,31^. In contrast, mice with the CBA background^31^, or F1(CBAxC57) mice^11,32^, retain normal hearing into adulthood. Therefore, the auditory analysis in mice was performed with both strains (Fig. 1k–n). The sounds in Fig. 1c–e generated excitation patterns that were around 20-35 dB above the hearing thresholds in the CBA/CaJ strain. Although the hearing thresholds in the C57BL/6 strain^33^ are much higher than the CBA/CaJ strain^25^, the excitation patterns still exceeded the hearing thresholds by around 10 dB in both Fig. 1i, m. Additionally, the excitation pattern of the FANTASIA sound was below the hearing thresholds at frequencies <20 kHz in both strains. The slightly elevated excitation pattern above 20 kHz may reflect the noise floor limit on the B&K 4191 microphone (Fig. 1f), rather than the real loudness of the system. Together, these analyses show our FANTASIA system, designed with a non-mechanical scanning mechanism and thorough acoustic treatments, can effectively reduce the noise floor of the imaging system to an exceptionally low level that has not been possible before. This very low noise background ensures the neuronal receptive fields we measured would not be affected acoustically by the imaging system.

### A simple, non-mechanical AOD scanning TPLSM system with flexible scanning modes

We designed our TPLSM system based on a single pair of AODs for non-mechanical scanning without acoustical noises while maintaining a simple architecture (Fig. 2a). A previously reported TPLSM system used a similar AOD pair to randomly access a subset of points in the 2D field of view (FOV) and traded spatial coverage for high speed for in vivo recording^34^. The same random-access pointing mode, although supported in our system (Fig. 2b and S2), is technically challenging when applied in awake animals where motion artifacts need to be minimized or corrected for^16,34^. An alternative strategy is to scan full frames at a fast speed, where motion correction algorithms would benefit from more continuous spatial and temporal information to recover the alignment^35^.

We designed our system with the following strategies to further support full-frame raster scanning modes. Linear frequency chirping was applied on both x- and y- AOD control signals (Fig. 2c). This led to a fast sweeping in deflected beam angle with a constant beam divergence on each axis^36–38^. Since the chirping speeds (frequency-changing slopes) on the two matched AODs were set equal, the resulting beam divergences would be identical on these axes and thus can be compensated simultaneously by a removable convex lens placed right after the AOD pair (Fig. 2a, c, e, f). We chose a divergence compensation lens (DvCL) with a focal length of 1000 mm (Fig. 2e). A chirping speed on the AOD was estimated at 30 MHz / 65.3 μs to get the beam after this DvCL collimated (also referred to as the nominal speed). While the chirping speeds were set equal on both axes, the frequency chirping range was set “one pixel” shorter on the y- axis AOD scan signal, thus resulting in a full-frame raster scanning at a speed of 23.5 frames per second (fps) that was within the video rate range. The frame rate can be further accelerated by using a DvCL with a shorter focal length (Fig. 1e), at the expense of increased width of a dark periphery of the FOV due to the necessary time to switch between scanning lines on the AODs (estimated ~13 μs, see also Materials and methods). Nevertheless, one may find a faster frame rate (e.g., 41.7 fps with an f = 750 mm DvCL, or 93.8 fps with an f = 500 mm DvCL) useful in recordings with a faster calcium sensor (e.g., jGCaMP8f^39^). Furthermore, the chirping speed of the AOD control signal can be set slower or faster than the nominal speed, controlling the beam after the DvCL converged or diverged, and therefore shifting the focal plane under the objective up or down from the nominal focal plane (Fig. 2d, g). This divergence control further enables a 3D multi-layer full-frame raster scanning mode (Fig. 2d, g, h). Figure 2f and h shows the exemplar images acquired by these newly added raster scanning modes.

Our system also features a simple dispersion compensation unit (DCU) based on a single reflection grating that is commercially available off-the-shelf (COTS). AODs can produce a significant amount of spatial dispersion that elongates the scanned point (Fig. 2i) and temporal dispersion that broadens the ultrafast laser pulses (Fig. 2j). To compensate for these dispersion effects from the AOD pair, a simple compensation scheme was reported^40^ to place a dispersive prism that produces the opposite amount of spatial dispersion before the AOD pair. The distance between the prism and the AOD pair can also be adjusted for the level of temporal dispersion compensated. The spatial dispersion and temporal dispersion can thus be simultaneously compensated by the DCU based on a single prism^34,38,40,41^. We further substituted this prism in the DCU with a COTS reflection grating which happened to have a blaze angle (5.4°) for optimal power throughput under our imaging condition (72%). Compared to previous DCU implementations, our grating-based DCU does not require customization on the prism^38^ and eliminated the need for several additional components (e.g., a half waveplate^34,38^, a cylindrical telescope^34^). Our simplified DCU implementation is easy to be aligned and features a lower inhomogeneity of estimated temporal dispersion across the beam (see Supplementary information). With this DCU, the elongated scanned points were largely compressed back to round (Fig. 2i). The laser pulse width was restored from 799.6 fs without compensation to 73.1 fs (the original pulse width is 65.4 fs). After the compensation, our system maintains comparably tight focus under both random-access pointing and raster scanning modes (Fig. 2k and S3). The estimated FWHMs (full widths half maximum), when imaged on 0.5-μm microbeads with a 10 × 0.5 NA objective, are 1.17 μm (lateral) and 6.38 μm (axial) under random-access pointing mode (*n* = 7 samples), and 1.45 μm (lateral) and 7.78 μm (axial) under fast raster scanning mode (*n* = 7 samples). The estimated FWHMs, when imaging on 0.2-μm microbeads with a 25 × 1.05 NA objective, are 0.56 μm (lateral) and 1.42 μm (axial) under fast raster scanning mode (*n* = 7 samples).

### Resolving neuronal structures with auditory functions in awake marmoset monkeys

**Fig. 3:**
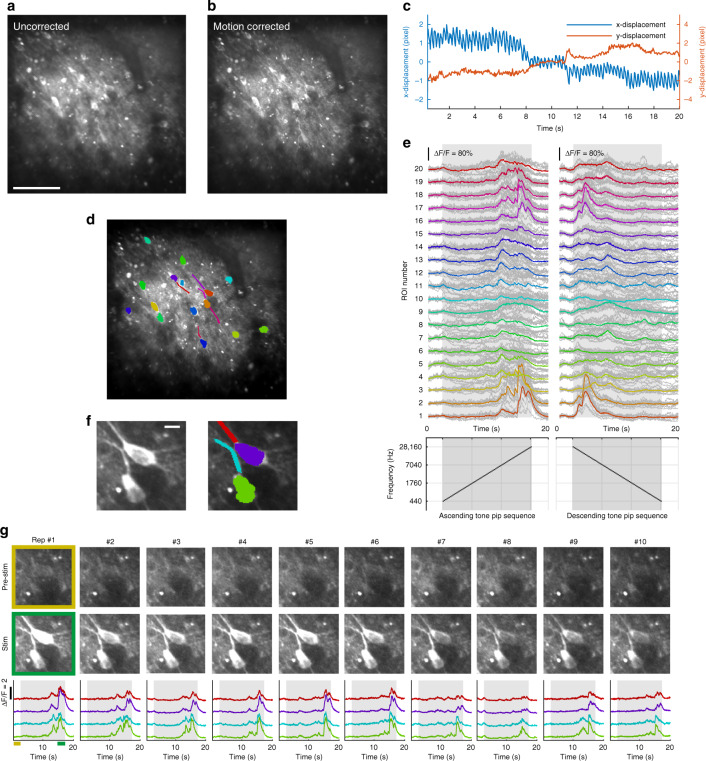
Imaging auditory cortical neurons in awake marmosets at subcellular resolution. **a** A two-photon image directly averaged across all frames within a 20-s trial. Scale bar: 100 μm. **b** An image averaged across the same trial with motion correction. **c** Estimated motion displacements along the x- and y- axes across the 20-second-long trial. **d** The exemplar ROIs of neuronal somas and processes in the FOV. **e** The calcium response traces of the ROIs labeled in (**d**) during the trials with both ascending (left) and descending (right) pure-tone pip sequences. The solid color lines indicate the mean response (*n* = 10 repetitions). The light gray lines indicate responses in individual trials. **f** The zoomed-in FOV of ROIs for two neuronal somas and two neuronal processes. Scale bar: 10 μm. **g** The individual-trial responses of the ROIs labeled in (**f**). The first row of fluorescence images shows the zoomed-in FOV during the pre-stimulus time. The second row shows the same zoomed-in FOV during the last 2.5 s of the stimulus presentation. The third row shows the response traces of the 4 ROIs following the same color scheme in (**f**). The integration time windows for deriving the images in the first two rows were indicated under the first panel in the third row

With our quiet and fast-scanning TPLSM system, we aimed to record neuronal auditory functions in awake marmoset monkeys through calcium signals labeled with GCaMP6s^42^. An exemplar FOV in the primary auditory cortex is shown in Fig. 3. A pure-tone pip sequence was played either ascending or descending in frequency at a moderate sound level (50 dB SPL) (Fig. 3e, lower panels) while imaging was performed at video rate under the 2D full-frame raster scanning mode. The resulted videos are shown in Supplementary Videos 1 and 2 at the original speed, with the original sound stimuli simultaneously presented. As expected, a population of neuronal somas was evident and responsive during the trials. Many subcellular structures, most likely neuronal processes, appeared also responsive during the trials. Moreover, motion blurring was evident when uncorrected raw images were directly averaged across time (Fig. 3a). This blurring was largely eliminated after a motion correction algorithm was applied^35^ (Fig. 3b). To quantify this motion, the estimated displacements along the x- and y- axes are plotted in Fig. 3c. This motion consisted of a fast component, presumably due to heart beating (around 4 Hz), and a slow shift in time. Together, the displacement amplitudes of the motion were largely within a range of 5 pixels on each axis (each pixel is 0.585 μm, the image is 653 by 652 pixels). The clear subcellular structures in Fig. 3b suggest the FOV was stably aligned after the motion correction. These data indicated that our imaging speed (23.5 fps) is effectively fast to preserve continuous spatial information in the tissue motion (see also Supplementary Videos 1 and 2).

Furthermore, for the exemplar regions of interest (ROIs) circled out in Fig. 3d, their response traces are shown in Fig. 3e. Most of the ROIs showed response peaks within the time window corresponding to frequencies of 7-20 kHz in both ascending and descending trials, suggesting a shared range of frequency selectivity by these neuronal structures. Additionally, to demonstrate the trial repeatability of these responses, the single-trial responses of several cellular and subcellular ROIs (Fig. 3f) are plotted in Fig. 3g. These responses were largely repeatable and robust across trial repetitions. Together, these data demonstrated the capacity of our imaging system to resolve functional activities in neuronal populations with subcellular resolution at video rate in awake marmosets.

### Auditory mapping of neuronal receptive fields with coverage of low sound levels

**Fig. 4:**
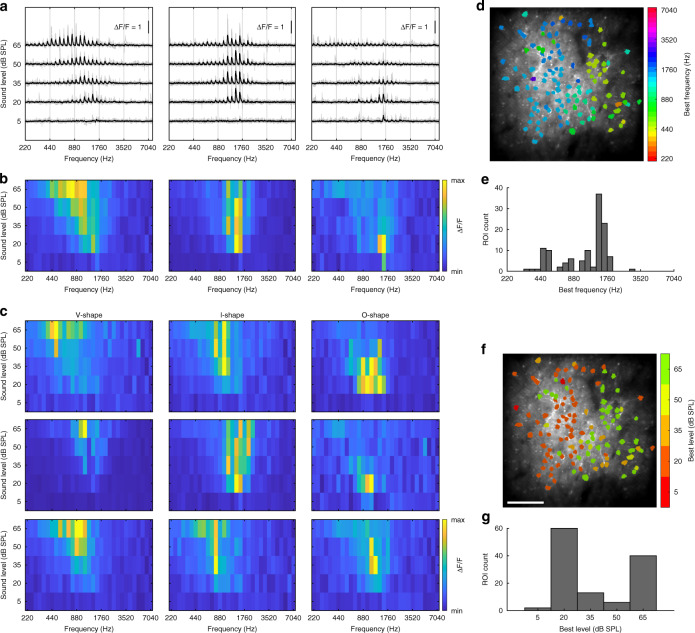
Receptive field mapping in auditory neurons with coverage of low sound levels. **a** Calcium traces of three exemplar neurons. Trials vary in pure-tone stimulus frequency and level. The experiment covers sound levels down to 5 dB SPL. Solid black lines: mean responses (*n* = 10 repetitions); light gray lines: responses of individual trials. **b** The FRA (frequency responsive area) summarized from the calcium responses in (**a**). These exemplar neurons show FRAs with the characteristic types of V-shape, I-shape, and O-shape. **c** More exemplar FRAs of these characteristic types from nine additional neurons. Among these exemplar FRAs. Clear responses at the sound level of 20 dB SPL are common. **d** The topography of neurons in the FOV labeled with their best frequencies (BFs). **e** The histogram of BF distribution. **f** The topography of neurons in the FOV labeled with their best levels (BLs). Scale bar: 100 μm. **g** The histogram of BL distribution

We further aimed to demonstrate our system’s capacity for measuring neuronal responses to sounds at low sound levels. A fundamental receptive field measure in the auditory system is to map the response of a neuron to pure tones of varying frequency and level (also known as its frequency response area, or FRA). Electrophysiology mapping of FRA routinely covers the sound levels near and below the hearing threshold^43^. In contrast, two-photon imaging studies usually missed covering this low sound level range, as many studies used sound levels starting from ~30-40 dB SPL^8,10,12,22,44^. Only a few went to the low sound levels (e.g., ~10-70 dB SPL in Issa et al 2014^6^, 0-70 dB SPL in Romero et al. 2019^11^). We performed FRA mapping in auditory cortical neurons with the sound level coverage down to the level of 5 dB SPL and frequencies spanning 5 octaves (Fig. 4).

The response traces of three neuronal ROIs were shown in Fig. 4a. These neurons varied in their sensitivities to frequency and level, but all had responsive trials at the sound level of 20 dB SPL. The evoked responses were further summarized in the FRA plots (Fig. 4b). The shapes of these FRAs fell into the previously described FRA categories:^43^ The first exemplar showed a classical V-shaped FRA that became stronger and wider at higher sound levels, while the second exemplar had an I-shaped FRA that maintained a similar width at all levels. Meanwhile, the third exemplar had an O-shaped FRA that featured a clear single peak at distinct frequency and level. More exemplars of these three FRA types were shown in Fig. 4c. These FRAs demonstrate the encoding of sound levels by the auditory neurons can be non-monotonic and complex. In each FRA, the best frequency (BF) and the best level (BL) were defined as the frequency and the sound level that elicited a maximal response. The BF and BL maps and histograms were plotted in Fig. 4d–g. Interestingly, many neurons had their best levels at ~20 dB SPL or lower, a phenomenon that has not been described in previous two-photon auditory studies^6–12,30,32,44^. These levels are also close to the species’ hearing threshold^17^. The lack of such a low and near-threshold best level in the previous two-photon studies is possibly due to the limit of the imaging system or the species studied. Nevertheless, the data here demonstrate our system’s capacity for measuring auditory responses at low sound levels and revealing auditory functions that were not reliably measurable before.

## Discussion

In summary, we have designed and built a two-photon imaging system that features an ultra-low acoustic noise floor during operation. This unprecedented quietness was achieved by a non-mechanical laser scanning mechanism with AOD scanners and acoustic treatments throughout the system. To facilitate recordings in awake animals, we added support for fast full-frame raster scanning with a cross pair of AODs originally designed for random-access scanning while maintaining the simplicity of the system architecture. In awake marmoset monkeys, stabilized FOV was achieved with motion correction algorithms that benefit from the speed of our full-frame scanning. Many neurons responded to pure tone stimuli with clear frequency sensitivity and repeatability. Moreover, the quietness of the system allowed reliable mapping of neuronal receptive fields down to the level of 5 dB SPL, an unmatched level in two-photon auditory studies. With this enhanced range of sound levels, many neurons with their best levels at or near the hearing threshold were revealed. Furthermore, we confirmed the noise floor of our two-photon system, when weighted with bandwidths of frequency selectivity, is near or lower than the hearing threshold in many species (e.g., marmosets, mice), further verifying our system’s capacity for performing auditory experiments with a clean acoustic background in species such as marmosets and mice.

As we have shown in Fig. 1, the acoustic noises of a TPLSM system may come from multiple parts of the laser system, or the mechanical scanner, or any mechanically moving device. Efforts have also been made in previous studies to reduce these noises, including keeping the laser’s power supply in a separate room^6,7^, enclosing the microscope and the experimental animal within a sound-attenuating box^8,10–12^, using sound-dampening materials and additional casing for the scanner^9,10^, and applying sinusoidal rather than sawtooth waveform to the galvo-scanner on the fast axis^45^. A further improvement for noises of the laser system is promising with the recently available fiber laser systems^46,47^. While delivering laser pulses comparable to those by Ti:Sapphire lasers, these systems are simpler in system architecture and have less demand on the power supply and cooling, leaving a possibility for further reduction in acoustic noise. To fully eliminate the laser scanning noise, a non-mechanical scanning mechanism is required. AOD scanning, as demonstrated in the current study, provides a unique opportunity to achieve full-frame raster scanning at video rate without acoustic artifacts. There may be other non-mechanical laser scanning mechanisms yet to be developed for silent two-photon imaging. Nevertheless, one needs to be careful of using mechanical scanners in auditory experiments. A thorough acoustic calibration is generally necessary to reveal potential spectral regions and levels that are vulnerable to acoustic interference.

To facilitate comparisons between auditory two-photon imaging studies, we propose a two-step procedure to report the acoustic noise floor of an imaging system (see also Supplementary information). (1) A raw acoustic power spectrum is helpful to reveal the physical peaks and spectral content of a noise (in dB SPL/Hz, as in Fig. 1c–f, an equivalent can also be Pascal/√Hz). (2) In addition to the acoustic spectrum, a species-specific excitation pattern can be plotted against the hearing threshold (both in dB SPL) to show the “perceptual” aspect of the noise floor in the testing species (as in Fig. 1g–n). Different species (and mice with different genetic backgrounds) vary in their hearing range and threshold levels^11,17,25,26,31,33^. It is thus important to perform a noise analysis with frequency- and species- dependent auditory properties specified. Further analysis examples in ferrets, rats, as well as mice with different tuning bandwidth measures, are given in Fig. S1. These analyses showed the major sound sources of a typical TPLSM system, but hardly our FANTASIA system would generate auditory excitation patterns exceeding the hearing threshold in a variety of species and strains commonly used in auditory research (Fig. S1). Together, we believe the demonstrated imaging strategies and noise analysis procedures in the current study shall help future two-photon auditory imaging studies with improved acoustic performance and data accuracy.

## Materials and methods

### Acoustic noise floor calibration and acoustic treatments

The acoustic noise floors of the imaging devices were measured by two calibration microphones. The Brüel & Kjær (B&K) type 4189 microphone has a claimed frequency range of 6.3 Hz–20 kHz and was installed on a sound level meter console (Brüel & Kjær type 2250). This system features a very low baseline noise floor (estimated as around -26 dB SPL/Hz @ 10 kHz based on the manufacturer’s data documents) for measuring very quiet sounds. The signal was amplified and conditioned with z-weighting (flat) by the console and output at a peak-to-peak level near but without saturation at a dynamic range of ±5 V. Another calibration microphone (B&K type 4191) has a claimed frequency range of 3.15 Hz–40 kHz and was connected to a customized amplifier with an output dynamic range of ±10 V. This system features a slightly higher noise floor (around -21 dB SPL/Hz @ 10 kHz based on the manufacturer’s data documents). The amplified signals were fed into analog input channels on a data acquisition (DAQ) card (National Instrument NI PCIe-6323) with the corresponding dynamic range settings. The signals were digitized at 16-bits precision, and a 200 kHz sampling rate. Each measurement lasted for one minute. The digitized signal was Fourier transformed and binned in the frequency domain to 1 Hz resolution to show the spectral density of the measured sound. To derive the absolute values in sound level, the spectral density was scaled based on the calibrated sensitivity of the measurement microphone and the amplification settings. The noise floors of the laser power supply and cooling units were measured at a one-meter distance away from the units. Other noise floors were measured by placing the microphone at the location of where the subject’s head is. To isolate the laser power supply and cooling units from the experimental subject, the laser console and the imaging system were placed inside a double-wall acoustic chamber (IAC acoustics, customized, ~3.4 m (W) x 3.1 m (D) x 2.6 m (H) in size), the interior of which was covered with 2” acoustic foam (Pinta, SONEX). An acoustic enclosure was customized to attenuate the noise from the laser console. The enclosure consisted of an inner layer of 1/2” sound absorber-barrier sheeting (McMaster, 54495T46), a middle layer of 1/4” acetal Delrin sheet (ePlastics, ACTLBLK0.25), and an outer layer of 2” acoustic foam (Pinta, SONEX). The auditory excitation patterns (Fig. 1) were estimated following previously described procedures^18^ using auditory tuning bandwidth data that were either measured behaviorally in marmosets^29^ or measured physiologically in mice^48^ (see also Supplementary information for generating excitation patterns in different species). For louder sounds that are clearly above the microphone noise floors, excitation patterns were derived based on the measurements with the B&K 4191 microphone, to better cover the higher frequencies. The FANTASIA imaging sound was so quiet that it reached the measurement noise floor on both microphones (Fig. 1f). Since the two microphones generally produced very similar spectra up to around 23 kHz on sounds at moderate levels (e.g., Fig. 1c, d), we combined the data from 4189 (below 23 kHz) and 4191 (above 23 kHz) measurements to derive the excitation pattern for the FANTASIA imaging sound.

### The system architecture of FANTASIA

The FANTASIA system was powered by a Ti:Sapphire ultrafast laser (Coherent Chameleon Vision S, with a 75 fs nominal pulse width at an 80 MHz repetition rate). The center wavelength was set as 920 nm. The laser beam was passed through an optical isolator (Thorlabs IO-5-TIS2-HP), a customized periscope to a raised platform from the main optical table, and then a beam expander (Special optics 56-30-2-8x-920). A reflection grating (Richardson Grating 53004BK01-500R, blaze angle = 5.4°) introduced spatial dispersion in the opposite direction of that introduced by the AOD pair. The inter-distance between the grating and AOD input aperture can be continuously adjusted by sliding a customized retroflector along a carrier rail to compensate for temporal dispersion. This DCU design was further discussed in the Supplementary Information. A half-wave plate (Newport, 10RP52-2B) was placed before the AODs to adjust the input polarization to the AODs. The laser scanning was controlled by a pair of AODs pre-mounted orthogonally to each other in a single enclosure (AA opto-electronic AA-DTSXY-A15-850). The input aperture of the AOD pair was fulfilled by adjusting the magnification on the beam expander. The AOD transition time was estimated as ~13 μs following the calculation convention^34,49^. The AOD pair enclosure was mounted 45° to the raised platform to match the spatial dispersion orientation with the compensation grating. An f = 1000 mm lens (Thorlabs AC508-1000-B-ML) was kinematically mounted right after the AOD pair when scanning mode was switched to any full-frame raster scanning mode to compensate for the beam divergence generated by the AODs. The scan lens (Thorlabs AC508-100-B-ML) and the tube lens (Thorlabs AC508-200-B-ML) relayed the beam to the objective. We used 10x objectives for in vivo imaging in marmosets due to their long working distances to accommodate our preparation (Olympus XLPLN10XSVMP, NA = 0.6, working distance=8 mm; Thorlabs TL10X-2P, NA = 0.5, working distance=7.77 mm). But a 25x objective was also tested (Olympus XLPLN25XWMP). The emitted photons were separated by a dichroic mirror (Semrock FF705-Di01-53*60) into a two-lens collection path (Thorlabs AC508-300-A-ML and ACL50832U-A) to maximize the use of the optical invariant of the PMT input aperture geometry (Hamamatsu H10769PA-40). An emission filter (Semrock FF01-520/70) was placed before the last collection lens to collect photons of the GCaMP emission range. The scanning pattern was generated on a NI PCIe-6356 card at 20 MHz to control a pair of direct digital synthesizers (AA opto-electronic DDSPA-B415b-0) that drove the AOD amplifiers (AA opto-electronic AMPA-B-34). The PMT signal was amplified by a modified amplifier (Becker & Hickl, PPA-100, the input impedance of the amplifier was modified as 500 Ω) and then digitized by a NI PCI-6115 at 10 MHz. The design and control code of FANTASIA are publicly available at https://github.com/x-song-x/FANTASIA. The spatial dispersion patterns (Fig. 2i) were images of random-accessed spots pointed on a fluorescence reference slide (Ted Pella, cat# 2273) captured by a wide-field camera. The laser pulse widths were measured with a correlator (FemtoChrome FR-103XL). The PSFs were measured with calibration slides prepared with 0.5-μm and 0.2-μm fluorescent microbeads (Thermo Fisher, T7284, T7280). FWHMs were calculated based on the Gaussian fitting of the PSF measures. The in vitro exemplar images were taken with a slide of fixed endothelial cells (Thermo Fisher, F36924).

### Marmoset preparation and in vivo two-photon imaging

The basic design of the marmoset chronic head-cap^50^ and cranial window^20^ implantation procedures have been described previously. An artificial dura pre-molded with silicone in a hat-like shape was implanted over the auditory cortex. Surgical silicone adhesive (WPI, Kwik-Sil) was filled to seal the gap between the craniotomy edge and the sidewall of the artificial dura. This artificial dura-based cranial window is removable for maintenance under anesthesia. AAV(DJ)-CaMKIIa-GCaMP6s (Stanford Vector Core) or a combination of AAV1-CaMKII0.4-cre (Penn Vector Core) and AAV(DJ)-EF1a-DIO-GCaMP6s (Stanford Vector Core) was injected into the cortex at a ~100 nL/minute speed. The CaMKII promoter was reported to control selective expression in cortical pyramidal neurons^51^. An optional coverslip was used to replace the center window part of the artificial dura and was secured and sealed to the silicone hat by Kwik-Sil. In vivo two-photon imaging was performed at least 3 weeks after viral injections. Subjects were habituated to sitting calmly in a semi-restrained chair following previously described adaptation procedures^50^. The animal was head-fixed and awake during the imaging sessions. The recording depths were ~200-300 μm below the pial surface. The laser power under the objective was typically kept ~30-100 mW. Two marmosets were used in the current study. Both animals were male and were 4-7 years old during testing. All experimental procedures conformed to local and US National Institutes of Health guidelines and were approved by the Johns Hopkins University Animal Use and Care Committee.

Two auditory experiments were performed. The first experiment (Fig. 3) consisted of two recording sessions, each with ten repetitions of a 20-second trial. A pure tone pip sequence was presented in the middle of the trial and was either ascending or descending in frequency in each session, respectively^20^. The sequence consisted of 73 pure tone pips, each pip with a 0.2-second duration and 20-millisecond sine ramps at both onset and offset. All pure tone pips were delivered at 50 dB SPL level, measured at the position of the animal’s head. The “ascending” sequence started with a pure tone of 440 Hz, continuing with each of the following pips ascending one semitone in frequency from the previous pip, and ending with a pure tone pip of 28160 Hz. The “descending” sequence was in the reversed order of the “ascending” sequence. The second experiment (Fig. 4) consisted of a recording session with ten cycles of 155 pseudo-randomized trials. Each of the 155 trials varied in frequency (out of 31 possible frequencies) and sound level (out of 5 possible sound levels). The frequencies ranged from 440 Hz to 7040 Hz, with a 2-semitone interval. The sound levels ranged from 5 dB SPL to 65 dB SPL, with a 15-dB interval. Each trial was 3-second long, with a 0.6-second pre-stimulus duration, a 0.6-second stimulus, and a 1.8-second post-stimulus duration. The 0.6-second stimulus consisted of three 0.2-second pure tone pips of the same frequency. Each pip had 20-millisecond sine ramps at both onset and offset. All sound stimuli were level controlled by a programmable attenuator (TDT, PA5) and delivered through a loudspeaker (KEF LS50) placed one meter away in front of the subject. Absolute sound pressure levels were calibrated by the measurement equipment mentioned above.

After the acquisition of images, motion correction was performed with NoRMCorre^35^ (non-rigid registration with a grid size of 128×128 pixels, resulting in 5×5 patches for each frame). All frames within a trial were first registered together, with a template image averaged across 10 frames in the middle of the trial. The averaged images of each trial were then registered together, after which the cross-trial shifts were applied back to all frames of each trial. After motion correction, cells were detected in a semi-automatic way with the CellMagicWand tool in ImageJ (https://github.com/fitzlab/CellMagicWand). We also subtracted neuropil contamination by extracting the fluorescence within a 5 pixel-wide ring surrounding each cell (excluding the cell pixels) and subtracting this value multiplied by a factor (0.4) from the cell’s raw fluorescence. The change in fluorescence ((*F*-*F0*)/*F0*) was calculated for each cell, in which *F0* is the mean fluorescence during the pre-stimulus time, *F* is the raw fluorescence value. The average change in fluorescence within the stimulus-presentation window was taken as a measurement of response amplitude to that stimulus.

## Supplementary information

Supplementary File

Supplementary Video 1

Supplementary Video 2

## Data Availability

The data that support the results within this paper are available from the corresponding authors upon reasonable request.

## References

[1] Ohki K (2005). Functional imaging with cellular resolution reveals precise micro-architecture in visual cortex. Nature.

[2] Sato TR (2007). The functional microarchitecture of the mouse barrel cortex. PLoS Biol..

[3] Dombeck DA, Graziano MS, Tank DW (2009). Functional clustering of neurons in motor cortex determined by cellular resolution imaging in awake behaving mice. J. Neurosci..

[4] Deneux T (2019). Context-dependent signaling of coincident auditory and visual events in primary visual cortex. eLife.

[5] Ceballo S (2019). Targeted cortical manipulation of auditory perception. Neuron.

[6] Issa JB (2014). Multiscale optical Ca^2+^ imaging of tonal organization in mouse auditory cortex. Neuron.

[7] Barnstedt O (2015). Functional microarchitecture of the mouse dorsal inferior colliculus revealed through in vivo two-photon calcium imaging. J. Neurosci..

[8] Panniello M (2018). Local and global spatial organization of interaural level difference and frequency preferences in auditory cortex. Cereb. Cortex.

[9] Tischbirek CH (2019). In vivo functional mapping of a cortical column at single-neuron resolution. Cell Rep..

[10] Xin Y (2019). Sensory-to-category transformation via dynamic reorganization of ensemble structures in mouse auditory cortex. Neuron.

[11] Romero S (2020). Cellular and widefield imaging of sound frequency organization in primary and higher order fields of the mouse auditory cortex. Cereb. Cortex.

[12] Gaucher Q (2020). Complexity of frequency receptive fields predicts tonotopic variability across species. eLife.

[13] Bullen A, Patel SS, Saggau P (1997). High-speed, random-access fluorescence microscopy: I. High-resolution optical recording with voltage-sensitive dyes and ion indicators. Biophysical J..

[14] Lechleiter JD, Lin DT, Sieneart I (2002). Multi-photon laser scanning microscopy using an acoustic optical deflector. Biophysical J..

[15] Nadella KMNS (2016). Random-access scanning microscopy for 3D imaging in awake behaving animals. Nat. Methods.

[16] Szalay G (2016). Fast 3D imaging of spine, dendritic, and neuronal assemblies in behaving animals. Neuron.

[17] Osmanski MS, Wang XQ (2011). Measurement of absolute auditory thresholds in the common marmoset (*Callithrix jacchus*). Hearing Res..

[18] Song XD (2016). Complex pitch perception mechanisms are shared by humans and a New World monkey. Proc. Natl. Acad. Sci. USA.

[19] Miller CT (2016). Marmosets: a neuroscientific model of human social behavior. Neuron.

[20] Song, X. D. et al. Functional maps of the primate cortex revealed by through-skull wide-field optical imaging. Preprint at 10.1101/2020.12.05.413047 (2020).

[21] Tani, T. et al. Sound frequency representation in the auditory cortex of the common marmoset visualized using optical intrinsic signal imaging. *eNeuro* **5**, ENEURO.0078-18.2018 (2018). 10.1523/ENEURO.0078-18.2018 PMC5937112 29736410

[22] Zeng HH (2019). Local homogeneity of tonotopic organization in the primary auditory cortex of marmosets. Proc. Natl Acad. Sci. USA.

[23] Moore BCJ, Glasberg BR (1996). A revision of Zwicker’s loudness model. Acust. U. Acta Acust..

[24] Osmanski MS (2016). Frequency discrimination in the common marmoset (*Callithrix jacchus*). Hearing Res..

[25] Radziwon KE (2009). Behaviorally measured audiograms and gap detection thresholds in CBA/CaJ mice. J. Comp. Physiol. A.

[26] Kelly JB, Kavanagh GL, Dalton JCH (1986). Hearing in the ferret (*Mustela putorius*): thresholds for pure tone detection. Hearing Res..

[27] Borg E (1982). Auditory thresholds in rats of different age and strain. A behavioral and electrophysiological study. Hearing Res..

[28] Zwicker E, Flottorp G, Stevens SS (1957). Critical band width in loudness summation. J. Acoustical Soc. Am..

[29] Osmanski MS, Song XD, Wang XQ (2013). The role of harmonic resolvability in pitch perception in a vocal nonhuman primate, the common marmoset (*Callithrix jacchus*). J. Neurosci..

[30] Bandyopadhyay S, Shamma SA, Kanold PO (2010). Dichotomy of functional organization in the mouse auditory cortex. Nat. Neurosci..

[31] Zheng QY, Johnson KR, Erway LC (1999). Assessment of hearing in 80 inbred strains of mice by ABR threshold analyses. Hearing Res..

[32] Bowen Z, Winkowski DE, Kanold PO (2020). Functional organization of mouse primary auditory cortex in adult C57BL/6 and F1 (CBAxC57) mice. Sci. Rep..

[33] Mikaelian DO, Warfield D, Norris O (1974). Genetic progressive hearing loss in the C57/M6 mouse: relation of behaviorial responses to cochlear anatomy. Acta Oto-laryngologica.

[34] Grewe BF (2010). High-speed in vivo calcium imaging reveals neuronal network activity with near-millisecond precision. Nat. Methods.

[35] Pnevmatikakis EA, Giovannucci A (2017). NoRMCorre: an online algorithm for piecewise rigid motion correction of calcium imaging data. J. Neurosci. Methods.

[36] Vučinić D, Sejnowski TJ (2007). A compact multiphoton 3D imaging system for recording fast neuronal activity. PLoS One.

[37] Chen XW (2011). Functional mapping of single spines in cortical neurons in vivo. Nature.

[38] Zheng T (2013). Visualization of brain circuits using two-photon fluorescence micro-optical sectioning tomography. Opt. Express.

[39] Zhang, Y. et al. jGCaMP8 fast genetically encoded calcium indicators. (2020). at 10.25378/janelia.13148243.

[40] Zeng SQ (2006). Simultaneous compensation for spatial and temporal dispersion of acousto-optical deflectors for two-dimensional scanning with a single prism. Opt. Lett..

[41] Zeng SQ (2007). Analysis of the dispersion compensation of acousto-optic deflectors used for multiphoton imaging. J. Biomed. Opt..

[42] Chen TW (2013). Ultrasensitive fluorescent proteins for imaging neuronal activity. Nature.

[43] Sadagopan S, Wang XQ (2008). Level invariant representation of sounds by populations of neurons in primary auditory cortex. J. Neurosci..

[44] Rothschild G, Nelken I, Mizrahi A (2010). Functional organization and population dynamics in the mouse primary auditory cortex. Nat. Neurosci..

[45] Wong AB, Borst JGG (2019). Tonotopic and non-auditory organization of the mouse dorsal inferior colliculus revealed by two-photon imaging. eLife.

[46] Dupriez P (2019). Ultrafast fiber lasers for multiphoton microscopy. PhotonicsViews.

[47] Hellerer, T. et al. 920 nm fiber laser delivering 100 fs pulses for nonlinear microscopy. Proceedings of SPIE 10082, Multiphoton Microscopy in the Biomedical Sciences XIX. San Francisco, California, United States: SPIE, 2019, 108820U, 10.1117/12.2507753.

[48] Taberner AM, Liberman MC (2005). Response properties of single auditory nerve fibers in the mouse. J. Neurophysiol..

[49] Otsu Y (2008). Optical monitoring of neuronal activity at high frame rate with a digital random-access multiphoton (RAMP) microscope. J. Neurosci. Methods.

[50] Gao LX, Wang XQ (2020). Intracellular neuronal recording in Awake nonhuman primates. Nat. Protoc..

[51] Dittgen T (2004). Lentivirus-based genetic manipulations of cortical neurons and their optical and electrophysiological monitoring in vivo. Proc. Natl Acad. Sci. USA.

