# Supplementary material for: A silent two-photon imaging system for studying in vivo auditory neuronal functions
Source: Light Sci Appl. 2022 Apr 14;11:96. doi: 10.1038/s41377-022-00783-y (PMC9010453; doi:10.1038/s41377-022-00783-y)
Supplement: Supplementary file 1 — Supplementary File [file 41377_2022_783_MOESM1_ESM.pdf]

Supplementary Information for

A silent two-photon imaging system for studying *in vivo* auditory neuronal functions

Xindong Song, Yueqi Guo, Chenggang Chen, and Xiaoqin Wang

Correspondence:
Xindong Song (songxindong@jhmi.edu) and Xiaoqin Wang (xiaoqin.wang@jhu.edu)

This pdf file includes:

Fig. S1, Auditory analysis of two-photon imaging sounds in marmosets, ferrets, mice, and rats.

Fig. S2, An exemplar image acquired by the system with a 25x 1.05 NA objective.

Fig. S3, Estimation of system's PSFs.

Supplementary Information

Further Supplementary Materials include:

Supplementary Video 1. A two-photon video of the ascending trial shown in Fig. 3e.

The video is played back at the original recording speed, with the original sound stimuli simultaneously presented. The video was motion-corrected and averaged across 10 repetitions.

Supplementary Video 2. A two-photon video of the descending trial shown in Fig. 3e.

The video is played back at the original recording speed, with the original sound stimuli simultaneously presented. The video was motion-corrected and averaged across 10 repetitions.

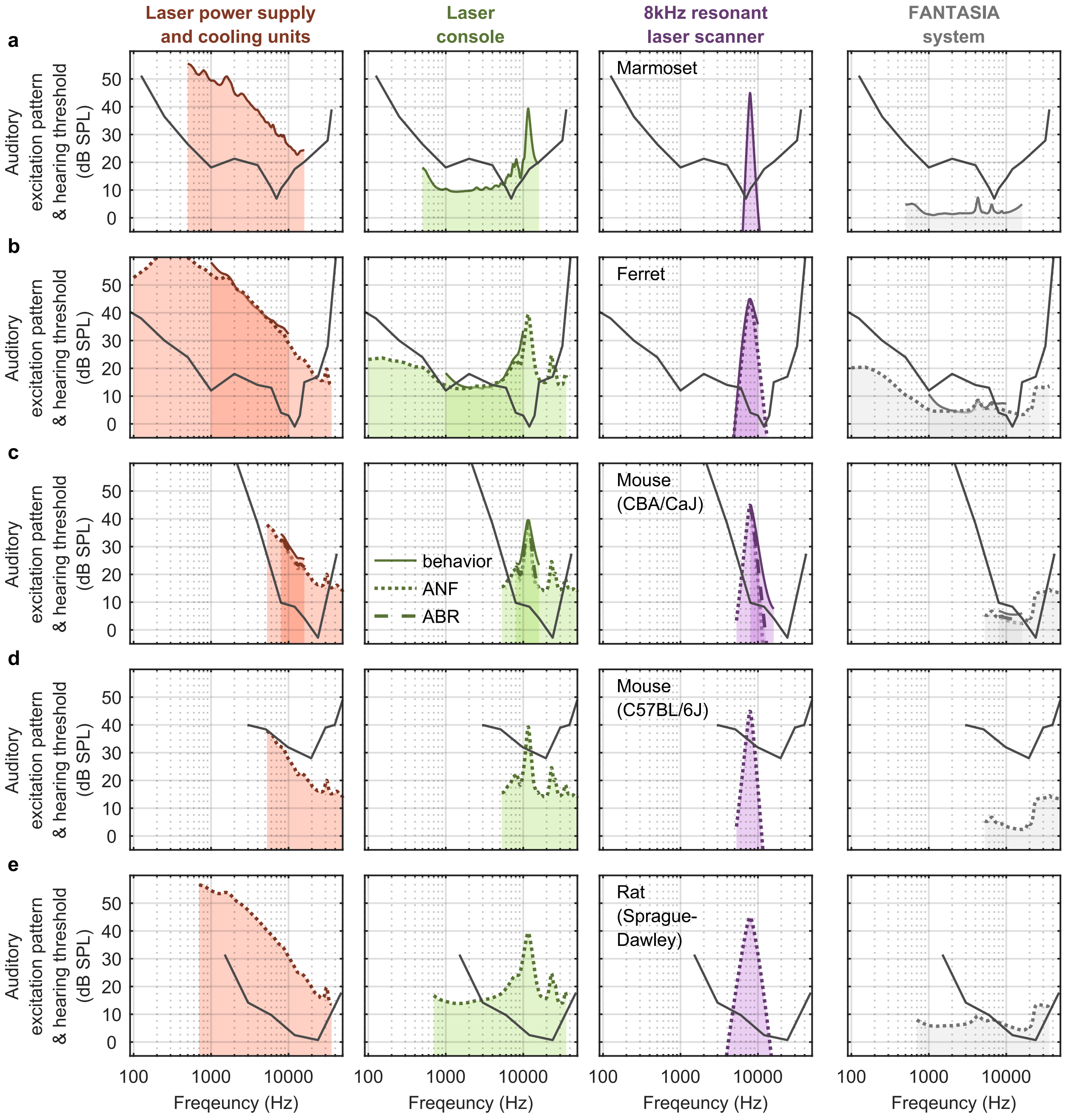

Fig. S1. Auditory analysis of two-photon imaging sounds in marmosets, ferrets, mice, and rats.
The auditory excitation patterns of the two-photon imaging sounds shown in Fig. 1c-f are estimated in various species and strains. These commonly seen species (strains) in auditory neuroscience studies include marmosets (**a**), ferrets (**b**), mice - CBA/CaJ strain (**c**), mice - C57BL/6 strain (**d**), and rats - Sprague-Dawley strain (**e**). The species-dependent auditory excitation patterns are plotted as the colored areas. The hearing thresholds are also plotted as the black solid curves for reference. Auditory excitation patterns are derived by integrating the noise spectra within the auditory tuning bandwidth at each frequency. These auditory tuning bandwidths were measured either behaviorally (colored solid lines), or by neural recordings from auditory nerve fibers (ANF, colored dotted lines) or auditory brainstem response (ABR, colored dashed lines). These different measures in the same species generally produce very similar excitation patterns on each sound analyzed here.

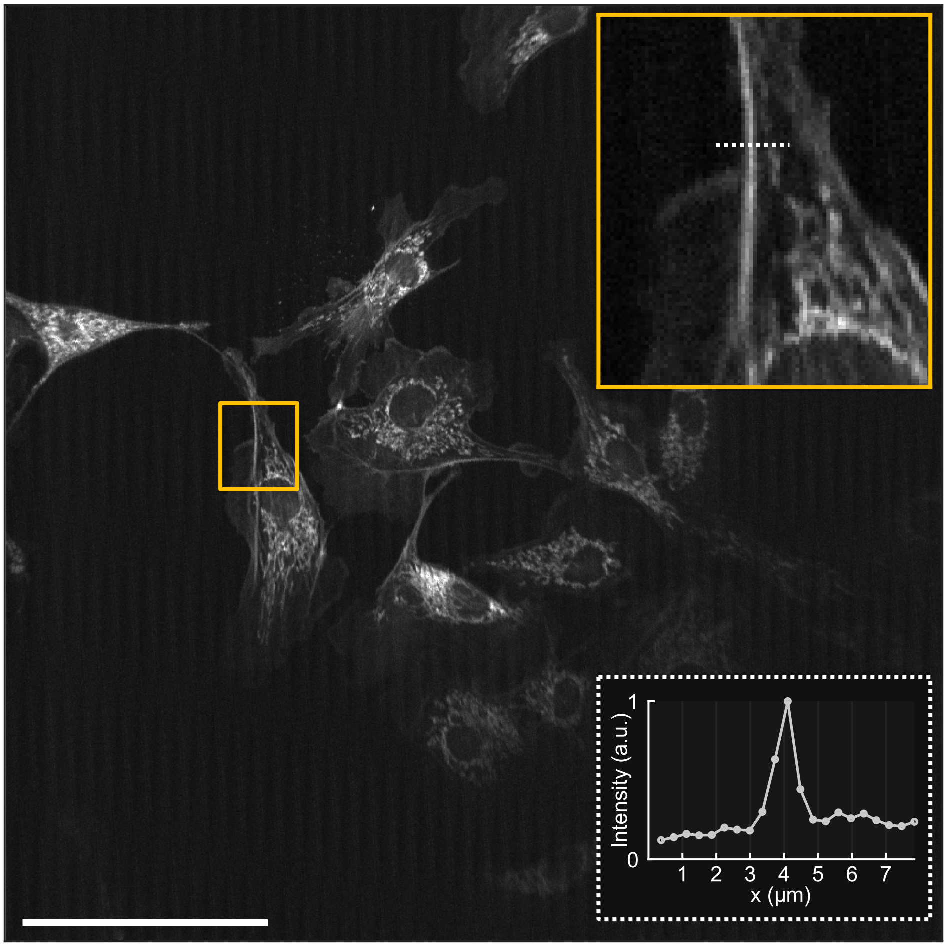

Fig. S2. An exemplar image acquired by the system with a 25x 1.05 NA objective.
A two-photon image of a fixed slide acquired under the 2D random-access pointing mode with a 25x objective (Olympus XLPLN25XWMP, NA=1.05, used in combination with an f=250 mm scan lens). A zoomed-in ROI is shown in the upper-right inlet. The intensity along the dashed line crossing over a thin fiber-like structure is further shown in the lower-right inlet. The FWHM of the structure is 0.86 μm, (each pixel is 0.373 μm). Scale bar: 100 μm.

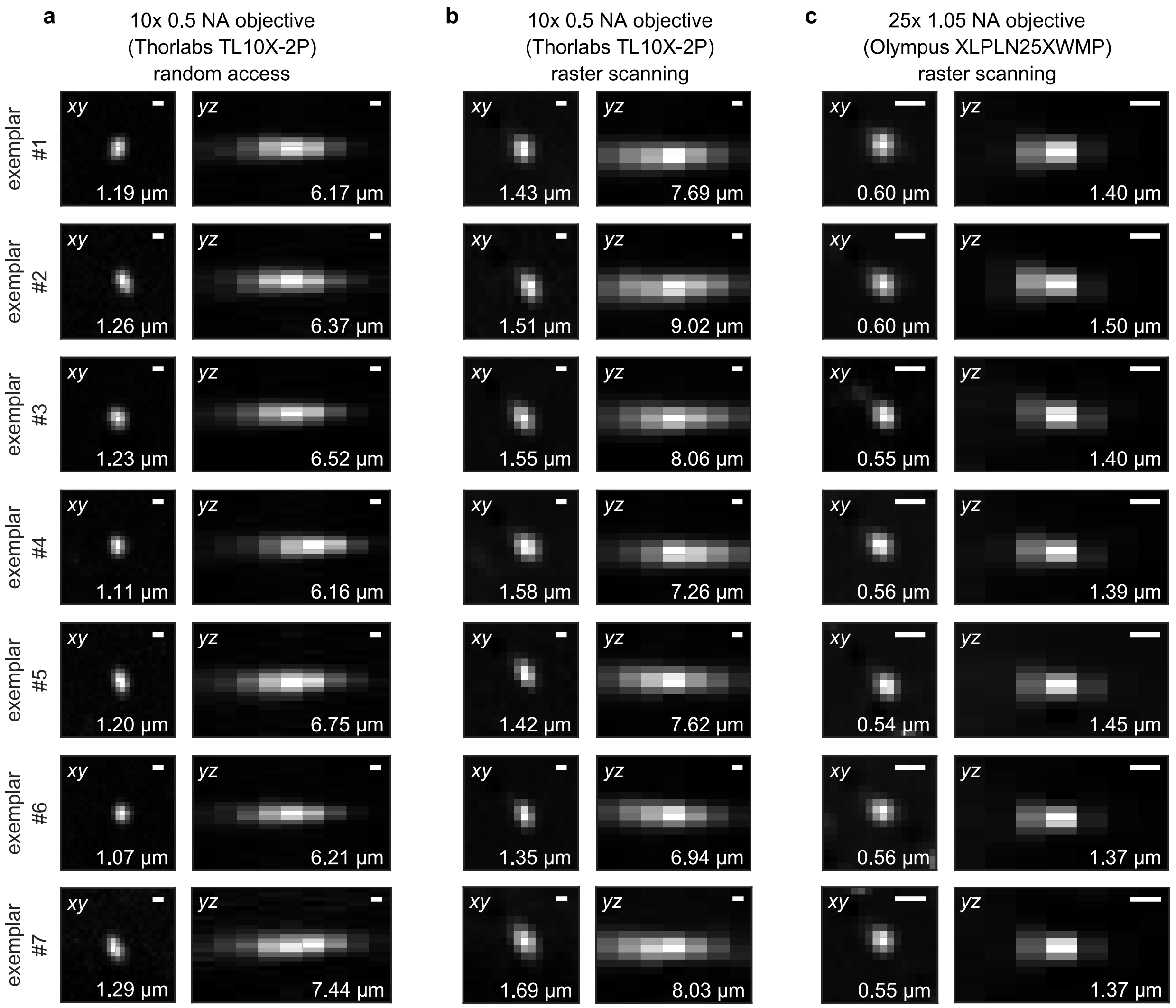

Fig. S3. Estimation of system's PSFs.

a. Exemplar PSFs measured on 0.5-µm microbeads under the Thorlabs 10x, 0.5 NA objective with the random-access scanning mode (n=7 samples). **b**. Exemplar PSFs measured on 0.5-µm microbeads under the Thorlabs 10x, 0.5 NA objective with the raster scanning mode (n=7 samples). **c**. Exemplar PSFs measured on 0.2-µm microbeads under the Olympus 25x, 1.05 NA objective with the raster scanning mode (n=7 samples). Scale bar: 1 µm. The numbers are FWHMs from Gaussian fitting.

Supplementary Information

Dispersion compensation unit (DCU) design

We designed our DCU to simultaneously compensate for spatial and temporal dispersion generated by the AOD pair. Our design was based on a previously described scheme[40]. This simple compensation scheme, in practice, has variations of using a single prism[34, 31, 40, 41] or an additional acousto-optic device[41] as the dispersive device placed before the AOD pair for compensation. We estimated our AOD pairs would generate spatial dispersion of 0.189 mrad nm^{-1} in the center of the FOV (acoustical velocity in the AODs: 650 m s^{-1}, center frequency for controlling the AODs: 87 MHz, laser wavelength: 920 nm, bandwidths of the AODs: 30 MHz, aperture: 12 mm). To choose among dispersive devices, we only consider commercial off-the-shelf (COTS) options, since our purpose is to build a simplified system to reduce the burden for auditory neuroscience labs to replicate. Other options, such as using another AO device (e.g., AOM) would significantly increase the system cost and thus is not suitable for our purpose. We list the following COTS options of the dispersive device in the table below.

	Prism<br>N-SF10 glass<br>60° Apex angle	Prism<br>N-SF11 glass<br>60° Apex angle	Reflection grating<br>150 grooves mm^{-1}<br>m = 1 (1st order)	Reflection grating<br>150 grooves mm^{-1}<br>m = -1 (-1st order)
Incident angle (°)	45.6	50.9	48.4	28.2
Output angle (°)	79.9	78.1	37.6	37.6
Optimal blaze angle (°)	NA	NA	5.4	4.7
COTS blaze angle (°)	NA	NA	5.4	4.7
Input angle range to generate spatial dispersion within the AOD FOV range (°)	~1.0	~1.5	~31	~23
Output aperture aspect ratio	3.6	2.9	0.84	1.1
GDD inhomogeneity across a 12mm aperture (fs^2)	~2100	~2700	~540	~410
COTS option (vendor, model)	Ealing<br>24-2966-000	Thorlabs<br>PS859	Richardson Gratings<br>53-*-500R	Richardson Gratings<br>53-*-501R

We have the following considerations in the DCU design. These considerations led us to choose a reflection grating instead of a prism to build the DCU.

One consideration is to reduce the number of additional components for optimizing laser power transmission. To assure maximal transmission, a prism-based implementation requires placing an additional half-wave plate in front of the prism to adjust the input polarization to the prism to optimize transmission efficiency[40, 34]. In contrast, a reflection-grating-based implementation only requires the blaze angle of the grating itself to be optimal for maximizing the diffracted beam power at the designated order. Interestingly, we found the COTS grating options that happen to have the theoretically optimal blaze angles for our application (5.4° or 4.7°). The 150 grooves mm^{-1}, 5.4° blaze angle grating resulted in a good power-transmission efficiency (72%) in our DCU and reduces the need for an extra half-wave plate.

Another consideration is to simplify aperture coupling in terms of controlling the ellipticity of the beam profile. For prism-based DCU implementations, due to the required geometry for dispersion

control, the output beam aperture leaving the prism can be significantly compressed in one direction (as the aspect ratio deviates from 1). In practice, an additional cylindrical telescope was added[34] to assure a round laser beam profile is fed to the AOD input aperture. Another option is to customize a prism[31] with an optimal apex angle to reduce the ellipticity of the output beam profile. Nevertheless, the additional components or costs of these options make them less desirable. In contrast, DCU implementations with the above-mentioned gratings would output a nearly round beam (aspect ratios as 0.84 or 1.1) and largely eliminate the need for additional components or customized devices to compensate for the ellipticity.

A third consideration is the ease of alignment. Comparing how sensitive the spatial dispersion is to the change in the incident beam angle to the compensation device, the grating-based options are generally more tolerant in alignment than prism-based options (see the table above). The entire spatial dispersion range of the AOD FOV corresponds to an incident angle range of ~1.0° and 1.5° for the prism options. In contrast, this incident angle range is ~23° and 31° for grating-based options, making the initial alignment and later maintenance less demanding and more robust.

A fourth consideration is to improve temporal dispersion inhomogeneity across the aperture. Per our estimation, the prism-based options introduce a temporal dispersion inhomogeneity across the AOD aperture at a scale of ~2100-2700 fs^2, whereas our grating-based option would only introduce ~410-540 fs^2. An increase in this dispersion inhomogeneity may further contribute to the widening of the laser pulse width.

Furthermore, we were able to design a retro-reflector slider between the grating and the AOD pair. This design allows easy and continuous control over how much temporal dispersion is compensated. Together, we implemented a simple, easy-to-align, and continuously adjustable dispersion compensation scheme based on a single reflection grating.

Estimating an auditory excitation pattern for a sound from its power spectrum density

To evaluate how a sound matters to a particular species' hearing, we convert the acoustic power spectrum density of the sound (in dB SPL Hz^{-1}) into a species-specific auditory excitation pattern (in dB SPL) to account for the bandwidth effect on loudness[28]. The resulting excitation pattern can thus be further compared to the species' hearing thresholds to reveal any potential frequency region affected by the sound (where the excitation pattern is significantly higher than the hearing threshold).

We borrowed a previously established human model and substitute the human parameters with corresponding animal parameters to generate the excitation pattern[S1, 23]. In summary, at a given center frequency, a rounded exponential filter was modeled to account for the bandwidth effect on how adjacent acoustic power is pooled together. The filter is expressed as $W(g) = (1 + pg)e^{-pg}$, where g is the deviation in frequency from the filter center frequency divided by the center frequency, p is 4 times center frequency divided by the equivalent rectangular bandwidth (ERB) of the filter, and $W(g)$ is the filter weight on acoustic power. Together, $W(f) = (1 + \frac{4(f-f_c)}{ERB})e^{-\frac{4(f-f_c)}{ERB}}$, where f is frequency, f_c is the center frequency. The ERB values have been measured behaviorally in several non-human mammalian species (e.g., mice[S2], marmosets[29], and ferrets[S3]). In some animal species, the ERB values can also be derived based on physiological measures (e.g., neural recordings in auditory nerve fibers[48, S4-S6], or auditory brainstem responses[S7]). One can

consider using such a physiological measure of frequency selectivity to calculate excitation patterns when the behavioral measure is not available.

It is also worth noting that different breeding strains of mice (e.g., CBA/CaJ, C57BL/6) may vary in their hearing thresholds due to the genetic background-associated hearing loss[33, 25, 11, 31]. The frequency selectivity, however, seems undifferentiable among tested strains when measured physiologically[48].

Supplementary references

1. Moore, B. C. J. & Glasberg, B. R. Suggested formulae for calculating auditory‐filter bandwidths and excitation patterns. *The Journal of the Acoustical Society of America* **74**, 750-753 (1983).
2. May, B. J., Kimar, S. & Prosen, C. A. Auditory filter shapes of CBA/CaJ mice: behavioral assessments. *The Journal of the Acoustical Society of America* **120**, 321-330 (2006).
3. Alves-Pinto, A. *et al*. Behavioural estimates of auditory filter widths in ferrets using notched-noise Maskers. *The Journal of the Acoustical Society of America* **139**, EL19-EL24 (2016).
4. Sumner, C. J. & Palmer, A. R. Auditory nerve fibre responses in the ferret. *European Journal of Neuroscience* **36**, 2428-2439 (2012).
5. Ruggero, M. A. & Temchin, A. N. Unexceptional sharpness of frequency tuning in the human cochlea. *Proceedings of the National Academy of Sciences of the United States of America* **102**, 18614-18619 (2005).
6. El Barbary, A. Auditory nerve of the normal and jaundiced rat. II. Frequency selectivity and two-tone rate suppression. *Hearing Research* **54**, 91-104 (1991).
7. Lina, I. A. & Lauer, A. M. Rapid measurement of auditory filter shape in mice using the auditory brainstem response and notched noise. *Hearing Research* **298**, 73-79 (2013).
